# Hereditary Influences on the Teeth

**Published:** 1867-09

**Authors:** Henry S. Chase

**Affiliations:** Iowa City


### HEREDITARY INFLUENCES ON THE TEETH.

BY HENRY S. CHASE, IOWA CITY.

Read before the Iowa State Dental Society, July, 1867.

THAT like produces like is a great natural law. Facts are too numerous and plain to doubt it. That there are seeming exceptions does not in the least disprove the assertion. Even when the progeny is not like the parents, it is still like a grandparent, uncle, or some other ancestor.

It is a wonderful thing that the germinal cell of an animal should be able, by cell multiplication, to make a being with all the organs and functions of the animal of which it is the type. Yes, this simple cell, with its nucleus and nucleolus, is the exact type of the animal which produced it, although the best microscope in the world would fail to discover to us, in its cell formation, the least resemblance to the animal which it yet truly represents in anatomical form, function, and often resemblance of features, and minute accidental or acquired peculiarities in body and mind.

Even after the spermatozoon has exercised its force on the ovum, the latter presents no different appearance under the microscope, from what it had before its fructification.

When this typical cell of the ovum is impressed by the vitality of its conjugal cell or spermatozoon, the new being may grow from the type of the father or the mother, or from both, according to the mutual impressions produced.

The inherent typical forms, residing in these cells of the male and female, are spiritual, magnetical, or at least imponderable and invisible. Each cell has its own peculiar power or *force*. If the force of the spermatozoon overcomes that of the ovum, then the new being will grow according to the prototype of the former; and so if the ovum cell has the strongest or most persistent force at the time of the conjunction of the two cells, then the new being will retain the type of the female. If the prototypes of these cells, or in other words, the father and the mother, are precisely alike in every respect except sex, then the new being will be precisely alike both of them, no matter what may have been the relative forces of the two at the time of their conjunction.

The most usual result proves a commingling or union of the two forces, in equal or different proportions, so that the progeny has some of its anatomical forms and functions like one parent, and the remainder like those of the other parent. A child may have the locomotive apparatus and the head like the father, and the vital apparatus like the mother, and so contrariwise.

Typical cells (ovum and spermatozoon,) are constantly subject to the magnetic influence of their prototypes. And this changes more or less with the changes taking place in the body and mind. And this magnetic influence has greater or less force on the cells, according to the strength of its persistence. Let us take a familiar example: A temperate man or woman becomes intoxicated for once only. The impression will have but little persistence. It will not have persistent force enough to change the typical magnetic form of the germinal cells. But let this person become a habitual drunkard, then the typical cell is so powerfully impressed by the prototype, that the magnetical form of the latter is impressed upon the germinal cell so powerfully, that it must in the act of reproduction make the new being with the depraved appetites of the parent This is the more surely the case when both parents are depraved. How often are even the peculiar idiotic features of the drunkard reproduced in offspring!

Beauties and defects then, of both body and mind, are sure to be reproduced in progeny. Observation has taught this independent of theory.

In our own vocation we see this daily exemplified in the teeth of our patients. We see it in form, location, quality, color, &c., &c. Let us take three generations, subject to precisely the same influences of climate, diet, hygiene, health, and marriage, and we shall find the dental organs all *precisely alike.* The child, father, and grandfather will have teeth all equally good. So on the maternal side.

But supposing the grandparents have good teeth, together with good health, habits, &c., &c. *Their* progeny will also have them by inheritance. Now if this second generation fall into bad habits of health, hygiene, diet, &c., the teeth become decayed, and if long remaining so, together with abnormal habits, then the typical cells of this generation will be permanently impressed with a depraved form, which will be sure, on reproduction, to cause the third generation to nherit and possess abnormal and defective dental organs. Now, by *reformation* of habits, in hygiene, diet, and health, this *third* generation can impress its typical cells so powerfully that it shall be able to transmit to the fourth generation a *better* if not a perfect Dental organization.

If one set of influences can degrade and destroy the teeth, an opposite set of influences can restore them to perfection in the next generation.

